# Cryptococcal meningitis presenting with recurrent syncope in a patient with chronic lymphoid leukemia: a case report

**DOI:** 10.1186/1757-1626-2-103

**Published:** 2009-01-29

**Authors:** Imdad Ahmed, Steven Powell, Michael Hoth, Ahmed Javed, Steffany K Moen, Melissa R Haehn

**Affiliations:** 1 Department of Hospital Medicine, Regions Hospital, University of Minnesota School of Medicine, Mail Stop 11107E, St. Paul, MN-55101, USA; 2 Department of Medicine, University of Minnesota School of Medicine, Minneapolis, MN-55455, USA; 3 Department of Psychiatry, Regions Hospital, St. Paul, MN-55101, USA; 4 Medical student, University of Minnesota School of Medicine, Minneapolis, MN-55455, USA

## Abstract

The clinical presentations of cryptococcal meningitis in HIV-negative patients may be different from that infected with HIV. We report a case of 75-year old male with chronic lymphoid leukemia presenting with recurrent syncope, bi-frontal headache and diplopia. This case discusses the atypical presentations of cryptococcal meningitis in HIV-negative patients and its importance of early diagnosis.

## Introduction

Among non-HIV patients with cryptococcal meningitis, most patients present with signs and symptoms of meningitis and fever is seen only in 50% of cases [1]. We have presented the first reported case of cryptococcal meningitis in a patient with chronic lymphoid leukemia (CLL) presenting with recurrent syncope.

## Case report

75 year old retired Caucasian male presented to the emergency room with an episode of syncope. He was also complaining of mild bi-frontal headache. His past medical history was significant for chronic myeloid leukemia and received second cycle of chemotherapy with Rituxan, Cytoxan and prednisone 2-weks prior to the ED visit. He was a nonsmoker and did not consume alcohol. He was 173 centimeter tall and weighed 68.7 kilogram. He was afebrile and laboratory investigations and chest X-ray were within normal limits. EKG showed normal sinus rhythm. A CT scan of the head showed acute sinusitis of bilateral maxillary sinus. He was diagnosed with acute sinusitis and discharged with oral levofloxacin.

One week later, he again presented to the emergency room with another episode of syncope for few seconds. MRI and MRA of the brain and MRA of the neck did not show any abnormal finding. A 2-D echocardiogram showed normal left ventricular function and no valvular abnormality. Serial cardiac markers were negative. No clear etiology of syncope was found and he was discharged home in a stable condition.

He again presented to the emergency room three days later with double vision of right eye, worsening bi-frontal headache and an episode of syncope. EEG was obtained and did not show any focal epileptic activity. Due to worsening bi-frontal headache and double vision, lumbar puncture was obtained. A cerebrospinal fluid(CSF) examination revealed clear and colorless fluid, an opening pressure of 25 cm of water, normal glucose and elevated protein at 72 mg/dl (normal: 15–45 mg/dl). His CSF white blood cell count was elevated at 38/mm^3 ^(normal: 0–5 mononuclear cells/mm^3^); 90%lymphocytes, 7% monocyte and 3% granulocytes. Cryptococci were detected in the CSF by the India ink staining, and CSF cryptococcal antigen titers were elevated. A serology for human immunodeficiency virus was negative. MRI of the head showed increased signal within convexity sulci in the FLAIR sequence, and there is increased leptomeningeal enhancement after IV gadolinium enhancement. Fundoiscopic evaluation did not show any choroidal involvement.

A diagnosis of cryptococcal meningitis was made and he was treated with intravenous amphotericin and oral flucytosine (induction therapy) for 4-weeks. At the time of the case report was written, the patient was being treated with oral fluconazole (consolidation therapy) and being discharged to a rehabilitation facility.

## Discussion

It is often difficult to reach a definitive diagnosis of cryptococcal meningitis in HIV-negative patients. Lumbar puncture, being an invasive procedure is not routinely performed in patients who presented with atypical signs and symptoms of meningitis. A study has shown that patients with an immunosuppressed condition, especially T-cell suppression, may present with less typical clinical manifestations of meningitis [2]. A blood cryptococal antigen test can be helpful which was positive in our patient. This can help physician to make decision to do lumbar puncture to exclude concurrent central nervous system infection. Delayed diagnosis is an important issue with this disease and reported a crude mortality of 19.1% in 94 cases of non-HIV-associated cryptococcal meningitis [2]. Our case was initially diagnosed with acute sinusitis and seizure. The recommended treatment is the same for patients with cryptococcal meningitis who do not have HIV infection, though no controlled trials have been done in this population [3].

In conclusion, although, headache, fever, lethargy, coma, personality changes, and memory loss are common clinical presentations of cryptococcal meningitis, it should be considered as differential diagnosis in HIV-negative immunocompromised patients such as systemic lupus erythematosus, chronic lymphoid leukemia, diabetes mellitus and renal transplant recipients presenting with atypical clinical features like recurrent syncope.

## Consent

Written informed consent was obtained from the patient's wife for publication of this case. A copy of the written consent is available for review by the Editor-in-Chief of this journal.

## Competing interests

The authors declare that they have no competing interests.

## Authors' contributions

IA did the literature search, analyzed and interpreted patient's data and wrote the manuscript. SP, MH, AJ, SKM and MRH did literature search, collected patient data and revised the manuscript for intellectual content. All authors read and approved the final manuscript.
